# Cutaneous head and neck melanoma in OPTiM, a randomized phase 3 trial of talimogene laherparepvec versus granulocyte‐macrophage colony‐stimulating factor for the treatment of unresected stage IIIB/IIIC/IV melanoma

**DOI:** 10.1002/hed.24522

**Published:** 2016-07-13

**Authors:** Robert H. I. Andtbacka, Sanjiv S. Agarwala, David W. Ollila, Sigrun Hallmeyer, Mohammed Milhem, Thomas Amatruda, John J. Nemunaitis, Kevin J. Harrington, Lisa Chen, Mark Shilkrut, Merrick Ross, Howard L. Kaufman

**Affiliations:** ^1^ University of Utah Huntsman Cancer InstituteSalt Lake CityUtah; ^2^ St. Luke's University Hospital and Temple UniversityPhiladelphiaPennsylvania; ^3^ University of North CarolinaChapel HillNorth Carolina; ^4^ Advocate Lutheran General HospitalPark RidgeIllinois; ^5^ University of Iowa Hospitals and ClinicsIowa CityIowa; ^6^ Minnesota OncologyFridleyMinnesota; ^7^ Mary Crowley Cancer Research CenterDallasTexas; ^8^ The Institute of Cancer Research/The Royal Marsden HospitalLondonUK; ^9^ Amgen, IncThousand OaksCalifornia; ^10^ The University of Texas MD Anderson Cancer CenterHoustonTexas; ^11^ Rutgers Cancer Institute of New JerseyRutgersNew Jersey

**Keywords:** cutaneous head and neck melanoma, talimogene laherparepvec, oncolytic virus, cancer immunotherapy

## Abstract

**Background:**

Cutaneous head and neck melanoma has poor outcomes and limited treatment options. In OPTiM, a phase 3 study in patients with unresectable stage IIIB/IIIC/IV melanoma, intralesional administration of the oncolytic virus talimogene laherparepvec improved durable response rate (DRR; continuous response ≥6 months) compared with subcutaneous granulocyte‐macrophage colony‐stimulating factor (GM‐CSF).

**Methods:**

Retrospective review of OPTiM identified patients with cutaneous head and neck melanoma given talimogene laherparepvec (*n* = 61) or GM‐CSF (*n* = 26). Outcomes were compared between talimogene laherparepvec and GM‐CSF treated patients with cutaneous head and neck melanoma.

**Results:**

DRR was higher for talimogene laherparepvec–treated patients than for GM‐CSF treated patients (36.1% vs 3.8%; *p* = .001). A total of 29.5% of patients had a complete response with talimogene laherparepvec versus 0% with GM‐CSF. Among talimogene laherparepvec–treated patients with a response, the probability of still being in response after 12 months was 73%. Median overall survival (OS) was 25.2 months for GM‐CSF and had not been reached with talimogene laherparepvec.

**Conclusion:**

Treatment with talimogene laherparepvec was associated with improved response and survival compared with GM‐CSF in patients with cutaneous head and neck melanoma. © 2016 Wiley Periodicals, Inc. *Head Neck* **38:** 1752–1758, 2016

## INTRODUCTION

Overall, 15% to 20% of cutaneous melanomas arise from head and neck locations despite this region representing <10% of total body surface area.^1^, ^2^, ^3^ Outcomes associated with cutaneous head and neck melanoma are poorer when compared with all other body sites, with a higher rate of recurrence and shorter disease‐free and overall survival (OS).^1^ Surgical treatment of cutaneous head and neck melanoma is technically challenging, owing to the difficulty in achieving appropriate margins in this cosmetically sensitive region.^4^, ^5^, ^6^ Because of the increased risk of recurrence and regional and systemic spread and recurrence with this location of melanoma, adjuvant therapy (including radiation therapy) is often used after surgical resection.^7^, ^8^, ^9^ For patients with unresectable head and neck disease, treatment options have been even more limited, with radiation therapy frequently used for locoregional disease control and palliation. Therefore, new treatment strategies are of high priority.

Oncolytic viruses are novel cancer treatments that mediate antitumor activity by selectively replicating in tumors and lysing tumor cells, subsequently releasing tumor‐derived antigens to promote antitumor immunity.^10^ Oncolytic viruses can be modified to express genes that further augment the antitumor immune response.^11^ Talimogene laherparepvec is a modified herpes simplex virus (HSV) type‐1 designed to specifically replicate in and lyse tumor cells.^12^ In addition to modifications designed to attenuate viral pathogenicity in normal tissues and to restore antigen presentation by HSV‐infected cells, talimogene laherparepvec is engineered to express the gene encoding human granulocyte‐macrophage colony‐stimulating factor (GM‐CSF).^12^ GM‐CSF can act to recruit and activate antigen‐presenting cells to process and present tumor‐derived antigens to help promote tumor specific T‐cell responses.^13^ Release of immune‐stimulatory viral proteins may further enhance the antitumor immune response.^11^ Responses in uninjected tumors, including visceral metastases, have been seen in patients treated with talimogene laherparepvec (in the OPTiM study responses to talimogene laherparepvec were observed in 34% of evaluable uninjected nonvisceral and 15% of evaluable visceral lesions),^14^, ^15^, ^16^, ^17^ indicating that an effective systemic antitumor response can be achieved.

In the randomized phase 3 OPTiM study, intralesional talimogene laherparepvec improved the primary endpoint of durable response rate (DRR; defined as complete response [CR] or partial response [PR] lasting continuously for ≥6 months) from 2% to 16% (*p* < .0001), compared to subcutaneous GM‐CSF in patients with stage IIIB/IIIC/IV melanoma that was not surgically resectable. The overall response rate (ORR), as evaluated by an independent Endpoint Assessment Committee, was also improved from 6% with GM‐CSF to 26% with talimogene laherparepvec (*p* < .0001, descriptive). Similarly, 11% of patients had a CR in the talimogene laherparepvec arm versus <1% in the GM‐CSF arm. Median OS with talimogene laherparepvec treatment was 23.3 months compared with 18.9 months with GM‐CSF treatment (hazard ratio [HR] = 0.79; 95% confidence interval [CI] = 0.62–1.00; *p* = .051).^16^ At the final planned analysis of OS, median OS was 23.3 months in the talimogene laherparepvec arm and 18.9 months in the GM‐CSF arm (HR = 0.79; 95% CI = 0.62–1.00; *p* = .049, descriptive]).^18^ This article describes a retrospective analysis of the subgroup of patients from the phase 3 OPTiM study who had cutaneous head and neck melanoma. DRR, ORR, time to treatment failure (TTF), and OS are reported to describe clinical outcomes with talimogene laherparepvec treatment in this melanoma subtype.

## PATIENTS AND METHODS

### Study design, patients, and treatment

Eligibility criteria and study design for the randomized, phase 3, open‐label multicenter OPTiM study are summarized in Supplementary Figure S1, online only, and have been reported in detail previously.^16^ Briefly, eligible patients were ≥18 years old with histologically confirmed cutaneous injectable and unresectable stage IIIB/IIIC/IV melanoma. Patients were excluded from the study if they had 3 or more visceral metastases, except lung metastases or nodal metastases associated with visceral organs, or visceral metastases >3 cm. This subgroup analysis included patients enrolled in the study who, at initial diagnosis, had melanoma located in the head and neck region (ie, scalp, face, and neck) as determined by the investigator. Patients were randomly assigned 2:1 to receive intralesional talimogene laherparepvec (≤4 mL initially at 10^6^ pfu/mL, then after 3 weeks 10^8^ pfu/mL once every 2 weeks) or subcutaneous GM‐CSF (125 μg/m^2^ daily for 14 days in 28‐day cycles). Discontinuation of study treatment because of disease progression was not required before 24 weeks unless alternate therapy was required or intolerance to treatment developed. All patients provided written informed consent, and all study procedures were approved by institutional review boards or ethics committees. The trial was registered with ClinicalTrials.gov (identifier NCT00769704).

DRR was the primary endpoint (defined as the rate of CR or PR lasting ≥6 months continuously and beginning within the first 12 months of treatment). Key secondary endpoints included OS (time from randomization to death), ORR, onset and duration of response, TTF (time from date of randomization to the date of the first clinically relevant progressive disease not followed by response or until death), and safety. Patients were evaluated clinically every treatment cycle (4 or 5 weeks) and/or radiographically every 12 weeks. DRR and ORR were determined using modified World Health Organization Criteria for Tumor Response Evaluation.^16^, ^19^ Patients with a best response of CR or PR per investigator assessment or who had received study treatment for ≥9 months were evaluated by an independent blinded endpoint assessment committee (EAC).

### Statistical analysis

Efficacy analyses were done for all patients with cutaneous head and neck melanoma who met the criteria for inclusion in this subgroup analysis and received at least 1 dose of study medication (see Patients above). All analyses were exploratory. The Fisher exact test was used to compare DRR and ORR between treatment arms. Time‐to‐event endpoints were evaluated using Cox proportional hazard models and unadjusted log‐rank tests. DRR and ORR were based on data from the primary DRR analysis; data cutoff for this analysis was December 21, 2012. OS and TTF analyses were based on data from the primary OS analysis, which was done after 290 survival events had occurred in the overall study population; the data cutoff date for this analysis was March 31, 2014. Multivariate analysis was conducted to adjust for imbalances in baseline prognostic factors. Statistical significance was interpreted at a two‐sided 5% confidence level without multiplicity adjustment.

## RESULTS

### Patient characteristics, disposition, and treatment

Of the 436 patients enrolled in the OPTiM study, retrospective review identified 87 patients (20%) with cutaneous head and neck melanoma (treated with talimogene laherparepvec, n = 61 [21%]; treated with GM‐CSF, n = 26 [18%]). The baseline clinical characteristics of these patients are shown in Table 1. Baseline demographics and characteristics for the intent‐to‐treat population are shown in Supplementary Table S1, online only. The median duration of follow‐up at the primary analysis of OS was 35 months (interquartile range [IQR], 13–43 months) for the talimogene laherparepvec group and 25 months (IQR, 13–39 months) for the GM‐CSF group.

**Table 1:** Baseline demographics and clinical characteristics.

	Talimogene laherparepvec	GM‐CSF
No. of patients	*N* = 61	*N* = 26
Median (IQR) age, y	70 (61–79)	66 (58–75)
Men, no. (%)	51 (84)	17 (65)
ECOG PS, no. (%)		
0	43 (70)	20 (77)
1	18 (30)	6 (23)
Disease stage at screening,^a^ no. (%)		
IIIB	9 (15)	5 (19)
IIIC	17 (28)	6 (23)
IVM1a	11 (18)	6 (23)
IVM1b	15 (25)	4 (15)
IVM1c	9 (15)	5 (19)
Elevated LDH, no. (%)	2 (3)	1 (4)
*BRAF* status,^b^ no. (%)		
Mutant	10 (16)	6 (23)
Wild‐type	6 (10)	4 (15)
Unknown/missing	45 (74)	16 (62)
Location of first recurrence,^c^ no. (%)		
Surgical scar (local)	17 (28)	4 (15)
In‐transit/satellitosis	21 (34)	7 (27)
Regional lymph node(s)	16 (26)	3 (12)
Distant skin site	7 (11)	6 (23)
Distant lymph node(s)	0	1 (4)
Visceral	3 (5)	2 (8)
Other	4 (7)	4 (15)
Missing	3 (5)	2 (8)
Median (IQR) time from initial diagnosis to first recurrence, y	0.6 (0.3–1.2)	0.5 (0.3–1.6)
Line of therapy, no. (%)		
First line	37 (61)	15 (58)
Second line or greater	24 (39)	11 (42)
HSV‐1 status, no. (%)		
Seropositive	38 (62)	13 (50)
Seronegative	18 (30)	13 (50)
Unknown	5 (8)	0

Abbreviations: GM‐CSF, granulocyte‐macrophage colony‐stimulating factor; IQR, interquartile range; ECOG PS, Eastern Cooperative Oncology Group performance status; LDH, lactate dehydrogenase; HSV‐1, herpes simplex virus type 1.

a Per case report form at screening.

b Because tissue was not collected prospectively, *BRAF* mutation analysis was reported by investigators and not evaluated centrally.

c Patients may have had more than one site of first recurrence. Site of first recurrence was evaluated at screening.

### Durable and overall response

DRR per EAC was 9.5‐times higher in the talimogene laherparepvec arm (36.1%; 95% CI = 24.2% to 49.4%) compared to the GM‐CSF arm (3.8%; 95% CI = 0.1% to 19.6%; *p* = .001). ORR was higher in the talimogene laherparepvec arm (47.5%; 95% CI = 34.6% to 60.7%) than in the GM‐CSF arm (7.7%; 95% CI = 1.0% to 25.1%; *p* = .0004). Eighteen patients (29.5%) in the talimogene laherparepvec arm had a CR, whereas no patient in the GM‐CSF arm had a CR. Eleven patients (18.0%) in the talimogene laherparepvec arm had a PR, compared with 2 patients (7.7%) in the GM‐CSF arm. DRRs and ORRs were more common among patients with disease stages IIIB, IIIC, and IVM1a (Supplementary Table S2, online only). Although ORR was numerically greater among patients with HSV‐seropositive disease (55.3%; 95% CI = 38.3–71.4) than patients with HSV‐seronegative disease (27.8%; 95% CI = 9.7–53.5), the difference between the 2 groups was not statistically significant (*p* = .14). Similarly, the DRR in patients with HSV‐seropositive disease (29.4%; 95% CI = 17.5–43.8) was numerically greater but not significantly different from that in patients with HSV‐seronegative disease (16.1%; 95% CI = 5.5–33.7; *p* = .20).

In the talimogene laherparepvec arm, responses were identified in 63.8% of injected lesions, 7.9% of uninjected nonvisceral lesions, and 10.8% of visceral lesions. Among 341 responding injected lesions, 311 (91.2%) were cutaneous or subcutaneous, and 29 (8.5%) were nodal; among 88 responding uninjected nonvisceral lesions, 65 (73.9%) were cutaneous or subcutaneous, and 6 (6.8%) were nodal.

**Figure 1:**
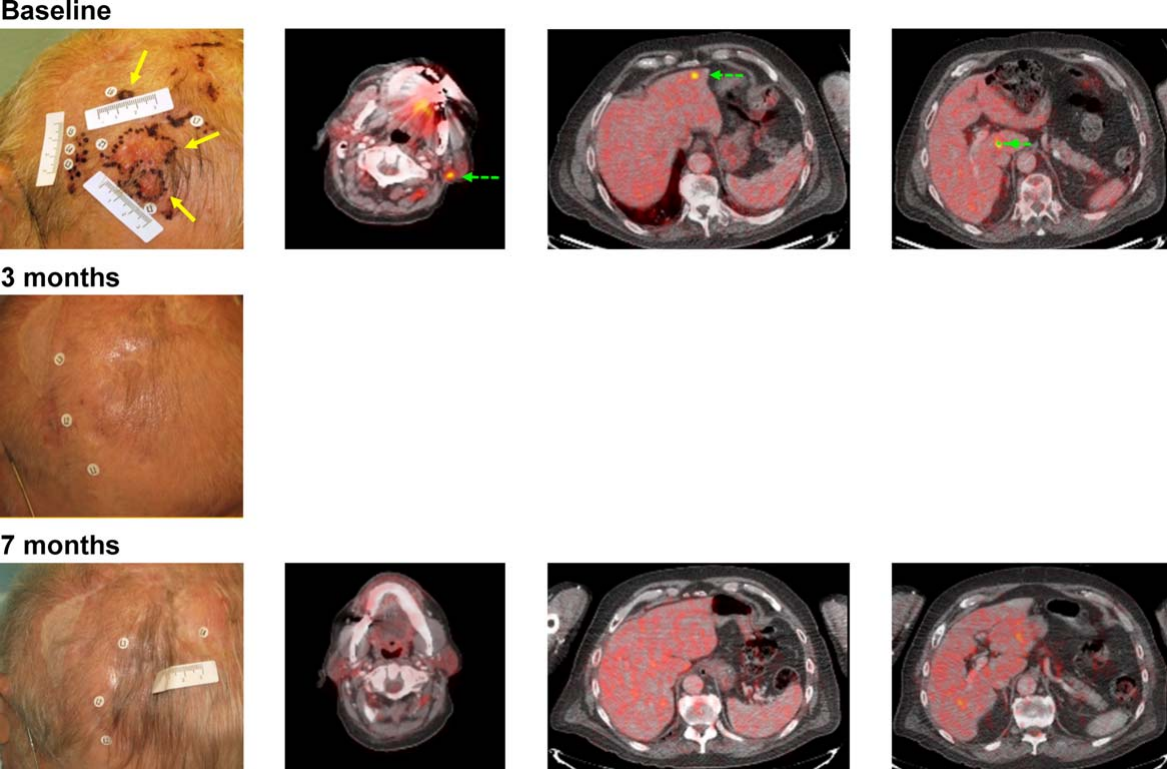
(A) Representative images from a patient with melanoma of the scalp with metastasis to cervical lymph nodes and liver (stage IVM1c). The patient was diagnosed 2 years before enrollment in OPTiM and had 2 surgeries: one at diagnosis, and another 1 year after recurrence. Top row: injection sites shown in yellow arrows at baseline (left panel). Uninjected sites are shown with green dashed arrows. Black dots mark tumor lesions. Sites included 1 fluorodeoxyglucose (FDG)‐avid left upper level V cervical lymph node (center left panel) and 2 FDG‐avid liver lesions (center right and right panels). Middle row: injections were stopped after complete resolution of scalp lesions after cycle 2 (1 cycle = 2 injections of talimogene laherparepvec). Bottom row: Complete resolution of cervical and liver tumors was documented by FDG‐PET CT at cycle 7. Patient was in complete response until the end of the trial, duration of response (complete response) was approximately 17 months.

**Figure 2:**
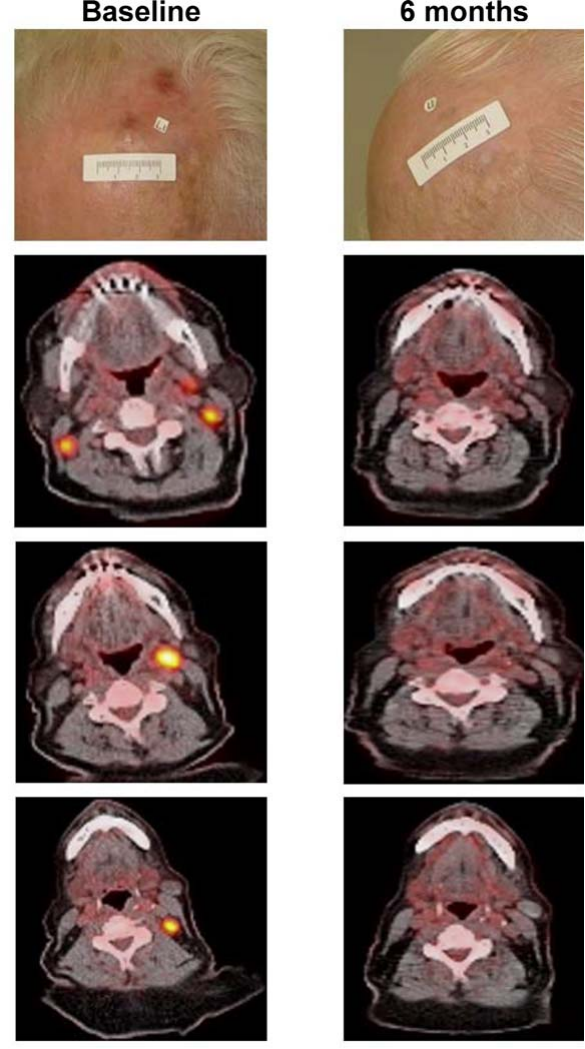
Representative images from a patient with stage IIIC disease randomized to talimogene laherparepvec who had a complete response. The patient was enrolled in the study with desmoplastic melanoma of the forehead with bilateral cervical fluorodeoxyglucose‐avid lymph nodes (left panel). Talimogene laherparepvec was injected only into the cutaneous lesion marked by the label (top row). At month 4, a partial response was reported and injection of talimogene laherparepvec was stopped. At cycle 6, a complete remission was reported that continued until the end of the study. Duration of response was 15.5 months. The patient was disease‐free at last follow‐up contact approximately 3 years after enrollment.

Photographs and radiographic images from representative patients with cutaneous head and neck melanoma who received treatment with talimogene laherparepvec are shown in Figures 1 and 2.

**Figure 3:**
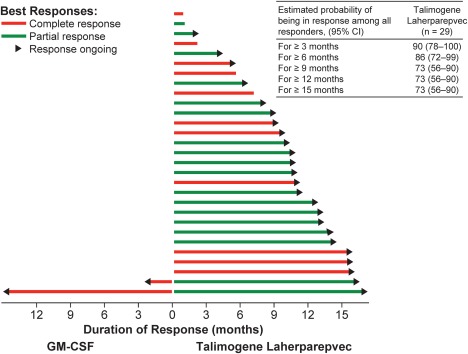
Duration of response for all patients with a response per endpoint assessment committee (EAC) was censored (marked by arrow) if at the last tumor assessment there was no evidence (per EAC) that the response had ended. Probability of being in response was estimated using the Kaplan–Meier method. Because only 1 patient in the granulocyte‐macrophage colony‐stimulating factor (GM‐CSF) group had a response lasting >3 months, probability of being in response was not calculated for this group.

Duration of response and probability of responders remaining in response at landmark time points are shown in Figure 3. Among patients in the talimogene laherparepvec arm with a response (*n* = 29), the estimated probability of being in response after 9 months was 73% (95% CI = 56% to 90%); this remained unchanged at the 12‐month and 15‐month time points.

### Time to treatment failure

**Figure 4:**
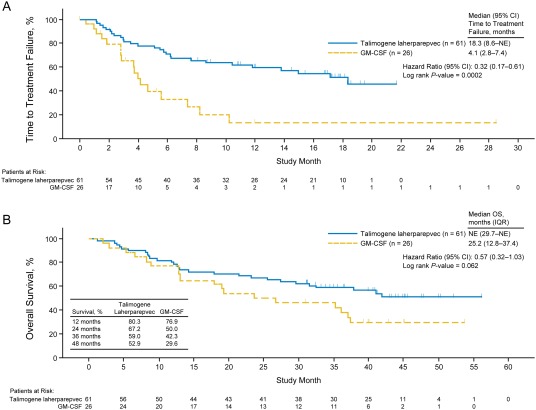
(A) Time to treatment failure per investigator assessment. (B) Overall survival. CI, confidence interval; GM‐CSF, granulocyte‐macrophage colony‐stimulating factor; IQR, interquartile range; NE, not estimable; OS, overall survival.

Median TTF was significantly prolonged for patients in the talimogene laherparepvec group (18.3 months [IQR, 8.6–not estimable]) compared with patients in the GM‐CSF group (4.1 months [IQR, 2.8–7.4]; HR = 0.32; 95% CI = 0.17–0.61; *p* = .0002). Kaplan–Meier curves for TTF are shown in Figure 4A.

### Overall survival and multivariate analysis

Kaplan–Meier curves for primary OS are shown in Figure 4B. Median OS was not estimable in the talimogene laherparepvec group (IQR, 29.7 months–not estimable) and was 25.2 months (IQR, 12.8–37.4 months) in the GM‐CSF group. The unadjusted HR for OS was 0.57 (95% CI = 0.32–1.03) favoring the talimogene laherparepvec group (unadjusted *p* = .062). At 24 and 48 months, estimated survival was 67.2% and 52.9%, respectively, in patients in the talimogene laherparepvec group and 50.0% and 29.6%, respectively, in patients in the GM‐CSF group. To adjust for potential clinically meaningful imbalances in prognostic factors of sex, disease stage, and Eastern Cooperative Oncology Group (ECOG) performance status, a multivariate sensitivity analysis was conducted. In this analysis, talimogene laherparepvec treatment was associated with improved OS compared to GM‐CSF (HR = 0.38; 95% CI = 0.20–0.72; *p* = .003; Table 2).

**Table 2:** Multivariate analysis of the effect of talimogene laherparepvec on overall survival.

Covariate^a^	HR (95% CI)	*p* value
Sex		
Female vs male	0.40 (0.18–0.89)	.025
ECOG PS		
0 vs 1	0.27 (0.14–0.53)	< .001
Disease stage		
IIIC vs IIIB	0.15 (0.04–0.55)	< .001
IV M1a vs IIIB	0.91 (0.35–2.41)	
IV M1b vs IIIB	2.07 (0.83–5.19)	
IV M1c vs IIIB	1.05 (0.39–2.87)	
Treatment		
Talimogene laherparepvec vs GM‐CSF	0.38 (0.20–0.72)	.003

Abbreviations: HR, hazard ratio; CI, confidence interval; ECOG PS, Eastern Cooperative Oncology Group performance status; GM‐CSF, granulocyte‐macrophage colony‐stimulating factor.

a Multivariate analysis includes prognostic covariates with imbalances at baseline.

## DISCUSSION

OPTiM was the first randomized, controlled, phase 3 study with an oncolytic virus to show therapeutic benefit in melanoma. The study met its primary endpoint, with the results indicating intralesional talimogene laherparepvec treatment improved DRR compared to subcutaneous GM‐CSF.^16^ This retrospective analysis of the OPTiM study evaluated clinical outcomes in the patients with cutaneous head and neck melanoma cohort and showed that talimogene laherparepvec demonstrated clinical benefit across different outcome measures in this difficult‐to‐treat subgroup.

Administration of talimogene laherparepvec was associated with higher DRR compared to GM‐CSF (36.1% vs 3.8%; *p* < .0001). In addition, responding patients had an estimated 73% probability of being in response 15 months or longer. As shown in the representative images (see Figure 1), some patients receiving talimogene laherparepvec had resolution of all lesions. The rate of CR (30%) was noteworthy. Achievement of CR is a particularly important consideration in patients with cutaneous head and neck melanoma because resection of these often cosmetically disfiguring lesions can be challenging, and some effective regional treatment options, such as isolated infusion/perfusion with antitumor agents, are not feasible for this anatomic site.^20^

Because retrospective comparisons in general can be flawed, particularly when comparing groups of patients that were not prospectively stratified, a multivariate sensitivity analysis that adjusted for imbalances in clinically important prognostic factors between the treatment arms in the cutaneous head and neck melanoma subgroup was performed. This analysis demonstrated a 62% lower risk of death in patients treated with talimogene laherparepvec compared with the GM‐CSF group (HR = 0.38; 95% CI = 0.20–0.72; *p* = .003). The median OS times in this retrospective analysis of the cutaneous head and neck melanoma subgroup are notable, and stand in contrast to previous reports that have noted poorer survival outcomes in patients with cutaneous head and neck melanoma.^1^ Importantly, treatment with talimogene laherparepvec has been associated with responses at uninjected tumor sites, including lesions in visceral organs,^14^, ^16^ indicating that a systemic antitumor response was initiated.

The better outcomes for patients with cutaneous head and neck melanoma compared with the overall study population are notable. One potential explanation for the better outcomes observed with talimogene laherparepvec in patients with cutaneous head and neck melanoma may be the higher proportion of patients with stage IIIB/IIIC disease than the overall study population (43% vs 30%). In an exploratory analysis of OPTiM, patients with stages IIIB/IIIC/IVM1a melanoma benefited the most from talimogene laherparepvec, with DRR as high as 33% for stages IIIB/IIIC and 16% for stage IVM1a, and median OS that was 41.1 months for patients with stage IIIB/IIIC/IVM1a disease in the talimogene laherparepvec arm compared to 21.5 months in the GM‐CSF arm (HR = 0.57; 95% CI = 0.40–0.80; *p* < .001 descriptive).^16^

Recently, a number of new immunotherapy and targeted therapy agents^21^, ^22^, ^23^, ^24^, ^25^, ^26^, ^27^ have been shown to be effective in patients with advanced melanoma but it is unclear what proportion of patients receiving these new therapies in these studies had cutaneous head and neck melanoma. Given its activity in patients with unresectable melanoma, its intralesional mode of administration, its ability to induce durable PRs and CRs, and responses at distant uninjected sites coupled with the prolonged TTF and OS, talimogene laherparepvec may represent a potential treatment option for patients with unresectable cutaneous head and neck melanoma. Notably, talimogene laherparepvec demonstrated a tolerable safety profile with most adverse events being within a spectrum of flu‐like symptoms, and generally transient and mild to moderate in severity.^16^

The key limitation of this study was its retrospective nature, which did not allow for control of clinical features across the treatment groups. As noted above, there were imbalances in duration of median follow‐up (1.4‐fold longer for patients treated with talimogene laherparepvec) and in baseline prognostic factors between arms that may have influenced the assessment of OS. It is also important to note that randomization of patients to treatment was not stratified by tumor location and that, although randomization in the overall population was 2:1 (talimogene laherparepvec:GM‐CSF), fewer patients with cutaneous head and neck melanoma were randomized to the GM‐CSF arm; the ratio in this analysis was 2.35:1. The influence on outcomes of this imbalance in randomization is uncertain.

In conclusion, in this retrospective analysis of the OPTiM study, administration of talimogene laherparepvec was associated with improved ORR, DRR, and OS compared to GM‐CSF in patients with cutaneous head and neck melanoma, consistent with results seen in the intent‐to‐treat population of the primary study.^16^ Talimogene laherparepvec is a potential novel treatment option for patients with regionally and distantly metastatic unresectable cutaneous head and neck melanoma.

## Supporting information

Supporting Information
