# Supplementary material for: Cutaneous head and neck melanoma in OPTiM, a randomized phase 3 trial of talimogene laherparepvec versus granulocyte‐macrophage colony‐stimulating factor for the treatment of unresected stage IIIB/IIIC/IV melanoma
Source: Head Neck. 2016 Jul 13;38(12):1752–8. doi: 10.1002/hed.24522 (PMC5129499; doi:10.1002/hed.24522)
Supplement: Supplementary file 1 — Supporting Information [file HED-38-1752-s001.docx]

**SUPPLEMENTARY APPENDIX**

**Supplementary Table 1. Baseline demographics and clinical characteristics of intent-to-treat population in OPTiM**

| **Characteristic** | **Talimogene laherparepvec arm***  **(n=295)** | **GM‑CSF arm^†^**  **(n=141)** |
| --- | --- | --- |
| Median (IQR) age, y | 63 (54–74) | 64 (54–74) |
| Men, n (%) | 173 (59) | 77 (55) |
| ECOG PS, ^‡^ n (%) |  |  |
| 0 | 209 (71) | 97 (69) |
| 1 | 82 (28) | 32 (23) |
| Disease stage at screening,^‡,§^ n (%) |  |  |
| IIIB | 22 (7) | 12 (9) |
| IIIC | 66 (22) | 31 (22) |
| IVM1a | 75 (25) | 43 (30) |
| IVM1b | 64 (22) | 26 (18) |
| IVM1c | 67 (23) | 29 (21) |
| Elevated LDH, n (%) | 15 (5) | 5 (3.5) |
| *BRAF* status,^\|\|^ n (%) |  |  |
| Mutant | 46 (16) | 23 (16) |
| Wild-type | 45 (15) | 23 (16) |
| Unknown/missing | 204 (69) | 95 (67) |
| Location of disease at initial diagnosis,^‡,¶^ n (%) |  |  |
| Head and neck | 61 (21) | 26 (18) |
| Chest, back, abdomen, pelvis | 72 (24) | 34 (24) |
| Hand, arm | 35 (12) | 23 (16) |
| Leg, foot, plantar, subungual | 112 (38) | 46 (34) |
| Other | 21 (7) | 6 (4) |
| Location of first recurrence,^‡,¶^ n (%) |  |  |
| Surgical scar (local) | 59 (20) | 26 (18) |
| In-transit/satellitosis | 108 (37) | 43 (30) |
| Regional lymph node(s) | 85 (29) | 31 (22) |
| Distant skin site | 26 (9) | 26 (18) |
| Distant lymph node(s) | 20 (7) | 7 (5) |
| Visceral | 22 (7) | 8 (6) |
| Other | 27 (9) | 10 (7) |
| Median (IQR) time from the initial diagnosis to first recurrence, year | 0.9 (0.4–2.2) | 1.1 (0.5–2.6) |
| Line of therapy, n (%) |  |  |
| First line | 138 (47) | 65 (46) |
| Second line or greater | 157 (53) | 76 (54) |
| HSV-1 status at baseline,^‡^ n (%) |  |  |
| Seropositive | 175 (59) | 78 (55) |
| Seronegative | 97 (33) | 45 (32) |

ECOG PS=Eastern Cooperative Oncology Group performance status; GM‑CSF=granulocyte-macrophage colony-stimulating factor; HSV-1=herpes simplex virus type 1; IQR=interquartile range; LDH=lactate dehydrogenase.

*4 patients were not treated with talimogene laherparepvec.

^†^11 patients were not treated with GM-CSF.

^‡^Contains unknown data.

^§^Per case report form at screening.

^||^ Because tissue was not collected retrospectively, BRAF mutation analysis reported by investigators and not evaluated centrally.

^¶^Patients may have had more than one site of first recurrence. Site of first recurrence was evaluated at screening.

**Supplementary Table 2. Overall (ORR) and durable response rate (DRR) by disease stage among patients who received talimogene laherparepvec**

| **Disease stage** | **N** | **ORR, n (%)** | **DRR , n (%)** |
| --- | --- | --- | --- |
| IIIB | 9 | 7 (77.8) | 6 (66.7) |
| IIIC | 17 | 12 (70.6) | 8 (47.1) |
| IVM1a | 11 | 5 (45.5) | 4 (36.4) |
| IVM1b | 15 | 2 (13.3) | 1 (6.7) |
| IVM1c | 9 | 3 (33.3) | 3 (33.3) |

**Supplementary Figure 1. Study design and treatment schema**

**
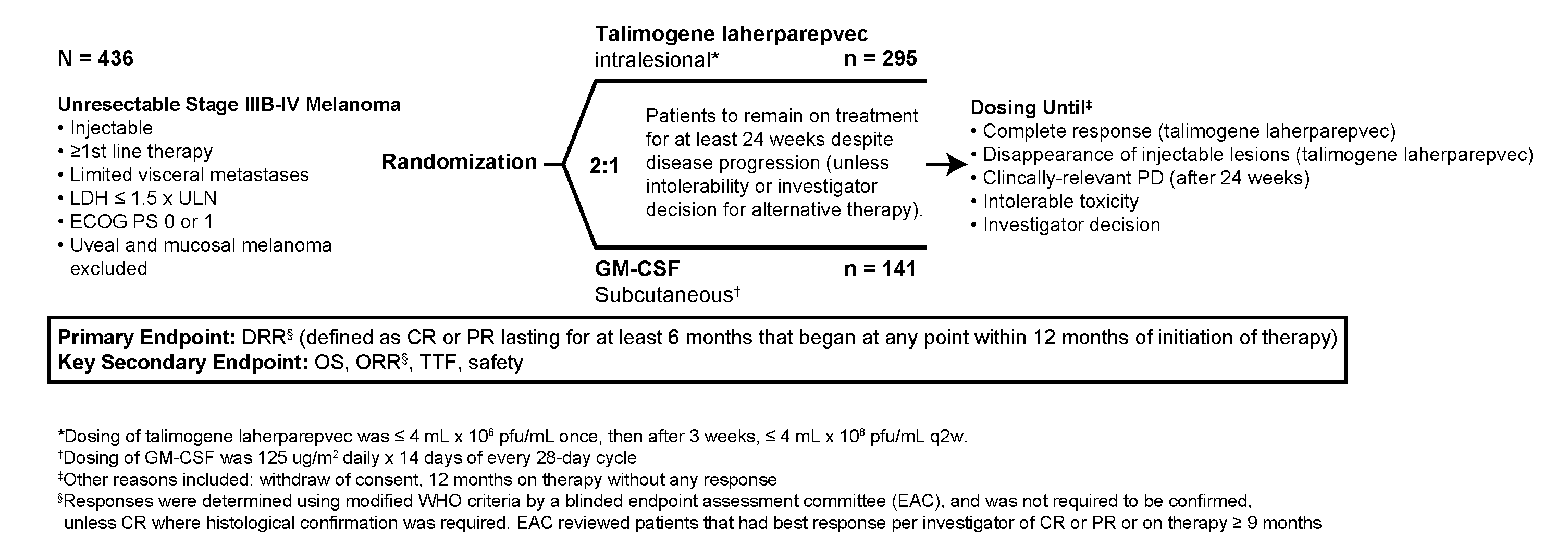
**

CR=complete response; DRR=durable response rate; EAC=endpoint assessment committee; ECOG PS=Eastern Cooperative Oncology Group performance status; GM‑CSF=granulocyte-macrophage colony-stimulating factor; LDH=lactate dehydrogenase; ORR=overall response rate; OS=overall survival; PD=progressive disease; PR=partial response; TTF=time to treatment failure; ULN=upper limit of normal; WHO=World Health Organization.
